# Economic valuation and determinants of informal care to disabled people with Chronic Obstructive Pulmonary Disease (COPD)

**DOI:** 10.1186/s12913-015-0759-6

**Published:** 2015-03-15

**Authors:** Luz María Peña-Longobardo, Juan Oliva-Moreno, Álvaro Hidalgo-Vega, Marc Miravitlles

**Affiliations:** Department of Economic Analysis, Seminar of Economics and Health, Faculty of Law and Social Sciences, University of Castilla-La Mancha, Cobertizo San Pedro Mártir s/n 45071, Toledo, Spain; Pneumology Department, Hospital Universitari Vall d’Hebron, Ciber de Enfermedades Respiratorias (CIBERES), Barcelona, Spain

**Keywords:** Economic value, Social costs, Informal care, Caregivers, Chronic Obstructive Pulmonary Disease

## Abstract

**Background:**

We aimed to estimate the monetary value of informal care of disabled people with chronic obstructive pulmonary disease (COPD) in Spain and to identify the main determinants of the time involved in informal caregiving.

**Methods:**

We used the Survey on Disabilities, Autonomy and Dependency carried out in Spain in 2008 to obtain information on disabled individuals with COPD and their informal caregivers. Assessment of informal caregiving time was performed using the proxy good method. A statistical multivariate analysis (ordered probit model) was performed to study the determinants of informal care provided.

**Results:**

It was estimated that 220,892 disabled people with COPD received informal care. The total annual number of caregiving hours was 694.44 million, with an estimated monetary value between 4,981 and 8,254 million EUR. Based on the condition of having received informal care, the cost of informal care per disabled person with COPD ranged from 24,549 to 40,681 EUR per year (depending on the shadow price applied). This value varies significantly depending on the degree of dependency; it ranged from 17,089 EUR per person annually for non-dependents to 33,033 EUR for those who were greatly dependent (under the most conservative scenario). Degree of dependency and the formal care received were the main variables that explained the variability of informal caregiving time provided.

**Conclusions:**

The results partially reveal the high hidden social costs, and the association between the level of dependency and the time provided by the caregivers. This information should be a useful tool to design policies that focus on improving caregivers’ well-being.

## Background

Chronic obstructive pulmonary disease (COPD) is a progressive disorder characterized by the occurrence of episodes of acute exacerbation with a high burden of morbidity and mortality. It is also a systemic and debilitating condition characterized by a progressive decline in the ability to perform basic activities of daily living (BADL) [1,2]. Furthermore, the frequency of exacerbations increases as the disease progresses to moderate and severe stages. It is associated with impaired physical, social, psychological and cognitive status in patients, namely, the quality of life related to health (HRQoL) [3].

The worldwide prevalence of COPD is estimated to be nearly 1% of the total population. Thus, it is estimated that COPD affects more than 52 million people and causes over 3 million deaths annually. In the United States in 2000, it was estimated that 10 million adults were diagnosed with COPD [4]. The “European Lung White Book” estimated that COPD prevalence in the adult population in Europe for the last two decades was 4–10%. The prevalence increases with age, reaching more than 10% in people aged 40 or older [5]. In Spain, the prevalence data are similar, with up to 9.1% of adults aged 40–70 years affected [6,7]. According to the EPI-SCAN study conducted in Spain, the prevalence for people aged 40–80 years was 10.2% (15.1% in males and 5.7% in females) [8].

The high prevalence, chronicity and frequent exacerbations of COPD cause frequent emergency room visits, hospitalizations, disability (severe core activity limitations) and death. COPD has thereby become one of the major chronic diseases in terms of its social, health and economic impact [9-11]. A large study of more than 1,500 primary care patients in Spain estimated the direct annual per patient costs of COPD as 1,760 USD, ranging from 1,484 USD for mild COPD to 2,911 USD for severe COPD. Overall, medication costs accounted for 40.8%, hospitalization costs for 43.8%, and medical visits and diagnostic tests for 15.4% [12] of costs. Izquierdo-Alonso et al. [13] estimated the annual medical costs of COPD to be 1,963 EUR in stage I, 2,842 EUR in stage II and 3,870 EUR in stage III. Medication costs accounted for 43.8%, 37.6% and 28.4% in each stage respectively [13]. With regard to the indirect costs of productivity losses, most studies consider that more than 60% of the total costs of the disease are explained by the indirect costs involved in premature death and disability [14].

However, despite the abundant literature about the direct costs of COPD, there is still a scarcity of studies that measure the costs of caregiving. Because of the disease’s chronicity and the often severe core activity limitations it causes, COPD patients usually require a high level of caregiving. Long-term care (LTC) expenditure has increased in recent decades in most OECD countries and is expected to rise further in coming years [15]. However, there is a significant variation in the burden of LTC among European countries, which can be explained by differences between formal long-term care systems and informal arrangements based mainly on caregiving provided by unpaid family members (informal care) [16].

Investigating the impact of informal care more broadly can provide valuable information that would be useful in considering how COPD is managed. Therefore, the main aim of this paper is to estimate the monetary value of informal care to people with COPD in Spain and to identify the main determinants of the time spent on informal care.

## Methods

### Data

Data were collected by the Survey on Disabilities, Personal Autonomy and Dependency Situations (EDAD-08) [17], conducted by the Spanish National Institute of Statistics. Due to the survey used in this paper, it was not necessary to perform an approval of an ethic committee. The data used here was from public source. The survey was divided into several questionnaires: a home questionnaire (first phase) and two subsequent individual questionnaires: a questionnaire on disabilities for individuals aged 6 and over and one on limitations for children aged 5 and under (second phase), as well as a questionnaire addressed to the primary caregiver.

Information collected between November 2007 and February 2008. The survey examined a representative sample of the disabled population at the national and regional levels. That means that the data used was not specific for COPD population but for disabled people suffering from several disabilities and different diseases. For this reason, we only selected those who recognized having suffered from COPD as the study focuses exclusively on this population. The sample studied included 96,000 Spanish households with disabled individuals, comprising 260,000 individuals who were initially interviewed. Of the total sample, 22,795 people with disabilities were identified and interviewed in depth. All the data were collected through personal interviews, and in exceptional cases, these data were complemented with telephone interviews. Among the variables included in the EDAD-08 survey are the personal characteristics of the people with disabilities, the characteristics of personal care and the characteristics of the setting in which the care was given. The National Institute of Statistics provides population weightings that allowed us to extrapolate data obtained in individual terms in the survey to a national scale. The economic assessment was done taking into account the population terms while the statistical analysis was done in individual terms.

Informal care is defined as attention from people who are not professional care workers provided to an individual with limited autonomy so that they can conduct one or more daily activities. Estimated informal care hours include caregiving provided by family members, friends or neighbors. Professional caregivers, from both the private and the public sectors, volunteers from non-profit organizations or domestic servants, and any caregiving or help provided in institutions or centers out of the home were excluded from the analysis. Likewise, caregivers who did not specify how much informal care they provided were also excluded. As a conservative criterion, we have censored the amount of care time to a maximum of 16 hours per day when the care time reported (18, 20 or even 24 hours) exceeded this figure.

Valuation of care time can be calculated using different methods [18-21]. As informal care is not offered commercially and thus there is no market price for it, it is necessary to allocate a shadow price for the valuation of these hours. There are two broad classes of valuation methods for the valuation of this time: (1) revealed preference methods including the opportunity cost and proxy good variants, and (2) stated preference methods. Given the characteristics of the information available in the EDAD survey, we choose the proxy good method for valuing care hours [21]. The proxy good method calculates time as an output. It then assigns a sum for the care provided by the informal caregiver on the assumption that if he/she were unable to provide the services, their tasks would have to be performed by another person. Thus, we considered the question of how much it would cost to substitute or replace the informal caregiver by hiring a professional caregiver.

Taking 2008 as a base year, two alternative scenarios were posited for the evaluation of informal care time. In the first scenario, the caregiving hours were valued using the price in the three Autonomous Communities (regions of Spain) with the lowest costs in the country. The sum used was 7.67 EUR per caregiving hour [22]. The second scenario then took into consideration the average national cost of public in-home care, i.e. 12.71 EUR per hour.

### Empirical model

A statistical analysis was carried out to estimate the marginal impact that a one-degree increase in the level of dependency would have on the hours of informal care (see [23,24]). In other words, starting with a person who suffers from COPD with disabilities—but without those disabilities constituting dependency at the level of the Official Scale in effect in Spain (non-dependent category)—the objective was to estimate how many hours of additional informal care that person would receive in the event that his/her condition was raised to a moderate degree of dependency, or to a state of severe or great dependency [25]. The Official Scale is based on a questionnaire as well as direct observation of the dependent candidate by a qualified professional. In the case of individuals with intellectual or cognitive impairment, an informed proxy respondent must answer the questionnaire. The determination of the degree of dependency takes into account medical reports and the use of prostheses. The Official Scale considers 47 tasks grouped into 10 activities (eating and drinking, control of physical needs, bathing and hygiene, other physical care, dressing and undressing, maintaining one’s health, mobility, moving inside the home, moving outside the home and housework). This scheme establishes four degrees of support: supervision (if the dependent only needs another person to prepare the necessary elements to perform the activity); partial physical attention (when the other person has to participate actively); maximum physical attention (if the person has to substitute for the dependent individual in performing the activity); and special attention (the dependent individual suffers behavior disorders that hinder performance of the task by the other person). The final score is the sum of the weights of the tasks for which the individual has difficulty, multiplied by the degree of supervision required and the weight assigned to that activity (for additional information, see [25]).

Given the asymmetry in the distribution of the number of hours of informal care, we designed an ordered probit model where the variable to be explained (number of care hours) fell into one of three categories (low, medium or high). This model allowed us to capture the ordinal nature of the independent variable, as well as the possibility of quantifying the intensity of such a variable (for examples, see [26-29]). The dependent variable is the number of hours of informal care provided weekly by a primary caretaker. It takes three different values: value 1 if the informal care does not exceed 30 hours per week (low); value 2 if the number of hours is between 31 and 85 (medium); and value 3 if the number exceeds 85 hours (high). The model’s specification is as follows:

$$ y*=\beta \hbox{'}X+\in, $$

where y* is not observed, X represents a vector of explanatory variables, β is a vector of the parameters and **ε** is the standard error. Moreover,

$$ \mathrm{y}=1\leftrightarrow \mathrm{y}*\le {\upmu}_1 $$

$$ \mathrm{y}=2\leftrightarrow {\upmu}_1<\mathrm{y}*\le {\upmu}_2 $$

$$ \mathrm{y}=3\leftrightarrow {\upmu}_2<\mathrm{y}*\le {\upmu}_3, $$

where μ refers to the parameters assigned to each category of order classified from 1 to 3; y = 1 is the low category of informal care hours; y = 2 is the medium category of informal care hours; and y = 3 is the high category of informal care hours.

More precisely, the functional form is as follows:

Annual informal care hours (primary caregiver)_i_ = β_0_ + β_1_ (age of the caregiver) + β_2_ (gender of the caregiver) + β_3_ (educational level of the caregiver) + β_4_ (degree of dependency of the person being cared for) + β_5_ (size of the municipality where the patient resides) + β_6_ (Autonomous Community where the patient resides) + β_7_ (formal in-home care received by the person being cared for) + β_8_ (formal out-of-home care received by the person being cared for) + β_9_ (whether caregivers reside at the same home) + u_t_.

## Results

According to the EDAD-08 survey, the number of disabled people aged 6 years or older living at home with one or more disabilities and with COPD totaled 461,884 (after applying the population weights provided by the survey; Table 1). Their mean age was 69.4 years and 57.5% were females. Almost 65% were classified as non-dependent, 14.5% as moderately dependent, 10.1% as severely dependent and 11% as greatly dependent. Furthermore, the most frequent disabilities are those to do with mobility (74.9%), housework (62.5%) and self-care (58.7%). The least frequent problems were those related to hearing (30.6%), vision (30.1%), communication (18.3%), learning (14.1%) and interaction (13.5%). On average, people with COPD present disabilities in three of the eight dimensions. In total, 66.3% of those with COPD received personal care because of their disability and 88.6% of these main caregivers were relatives or friends (informal care). Of the total informal caregivers, 76.1% were females with an average age of 56.5 years.

**Table 1:** **Characteristics of patients with COPD and their informal caregivers**

	**Disabled people with COPD (n = 461,884)** ^(*)^	**Disabled people with COPD who received Informal care (n = 220,892)** ^(*)^	**Primary informal caregivers** ^(**)^ **(n = 220,892)** ^(*)^
	**Average (SD) or percentage**		
**Age**	69.4 (17.1)	72 (18.9)	56.5 (14.9)
**Gender**			
Male	42.5	41.8	23.9
Female	57.5	58.2	76.1
**Marital Status**			
Married	66.8	48.7	69.3
Single	19.6	13.1	18.2
Widowed	7.8	35.9	6.2
Separated/divorced	5.8	2.3	6.3
**Educational level**			
Illiterate or primary school incomplete	44.9	59.1	29.2
Primary or equivalent	29.3	27.4	33.9
Secondary school/Middle level professional	20.3	11.3	30.1
University degree or equivalent	5.5	2.2	6.8
**Level of dependency**			-
Non-dependent	64.4	33.6	-
Moderately dependent	14.5	25.9	-
Severely dependent	10.1	18.7	-
Greatly dependent	11.0	21.8	-
**Average hours of care giving provided per day**			
Non-dependent			6.7 (8.2***)
Moderately dependent			8.9 (11.7***)
Severely dependent			11.9 (15.6***)
Greatly dependent			12.7 (17.0***)

Source: EDAD 08 Survey. *Data extrapolated to entire population with COPD**Caregivers providing at least 1 hour of care per day. ***Daily hours without restriction.

Based on the condition of having received informal care, the average number of hours of informal care per day was 9.58. This figure was strongly correlated with the severity of the disability and the corresponding dependency, ranging from 12.74 hours per day for those who were greatly dependent to 6.73 for non-dependency (according to the Official Scale). In total, 87.7% of caregivers provided care 6 or 7 days per week. Regarding experience, 43.4% of caregivers had provided care for more than 8 years, 20.0% had between 4 and 8 years’ experience, 29.4% had less than 4 years’ and 7.0% did not answer (Table 1).

The average number of estimated hours of care was 3,346.2 annually and the monetary valuation of those hours ranged from 24,549 to 40,681 EUR per person annually (Table 2). This value could vary significantly depending on the degree of dependency (Table 2). Thus, under the most conservative scenario, the monetary value ranges from 17,089 EUR per person annually when caregivers care for non-dependents to 33,033 EUR for greatly dependent patients. Likewise, under the second scenario the monetary value ranged from 28,318 for caregivers caring for non-dependents to 54,740 for greatly dependent patients. The total number of hours of care provided annually in Spain for disabled people with COPD is 694.44 million and the monetary value of these ranges from 4,981 to 8,254 million EUR (scenarios 1 and 2 respectively).

**Table 2:** **Value of informal care (main caregivers) by degree of dependency, proxy good method**

	**Non-dependents**	**Moderately dependent**	**Severely dependent**	**Greatly dependent**	**Total**
Monetary value. First scenario (SD)	17,089.38 (14,451.76)	22,833.99 (14,827.18)	30,429.68 (13,182.81)	33,033.68 (12,087.48)	24,549.82 (15,255.84)
Monetary value. Second scenario (SD)	28,318.91 (23,948.10)	37,838.32 (24,570.21)	50,425.19 (21,845.31)	54,740.30 (20,030.23)	40,681.64 (25,280.55)

Source: EDAD-08.

Average cost per year.

The statistical analysis highlights the importance of the degree of dependence when explaining the differences in the amount of care time provided by caregivers (Table 3). The results are interpreted as follows: relative to the reference category (non-dependent), a primary informal caregiver of an individual in a category of heavy dependence is 44.3% more likely to fall into the high-level category of care, 13.5% less likely to be in the medium-level category, and 30.8% less likely to fall into the low-level category. The results of a primary informal caregiver of an individual in a category of moderate dependence or severe dependence, relative to the reference category, would be interpreted the same way. Another significant variable associated with the number of hours received by disabled people with COPD was formal in-home care (e.g. telecare, home help) and formal out-of-home care (e.g. care homes, day centers). Thus, patients who received formal in-home care were 13.6% more likely to receive a high amount of caregiving hours than those who did not receive formal in-home care. However, formal out-of-home care was not statistically significant and did not explain the probability of caregiving received. These results suggest that in patients with COPD, informal care is complementary to formal in-home care services (Table 3).

**Table 3:** **Determinants of providing informal care giving hours**

	**High care giving hours**		**Medium care giving hours**		**Low care giving hours**	
	**Marginal effect (SD)**	**P**	**Marginal effect(SD)**	**p**	**Marginal effect(SD)**	**P**
**Moderate dependence**	0.141 (0.033)	<0.001***	−0.023 (0.008)	0.007***	−0.118 (0.025)	<0.001***
**Severe dependence**	0.329 (0.035)	<0.001***	−0.091 (0.017)	<0.001***	−0.238 (0.021)	<0.001***
**Heavy dependence**	0.443 (0.032)	<0.001***	−0.135 (0.018)	<0.001***	−0.308 (0.019)	<0.001***
**Formal care in-home**	0.136 (0.047)	0.004***	−0.027 (0.014)	0.064*	−0.108 (0.032)	0.001***
**Formal care out-of-home**	−0.026 (0.034)	0.450	0.001 (0.001)	0.307	0.024 (0.033)	0.461
**N**						1298
**LR chi2**						370.52
**Prob > chi2**						0.0000
**Pseudo R2**						0.1374

Results of the ordered probit model.

Significance level at 99% (***) and 90% (*). Dependent variable: Annual caregiving hours. Control variables: age, gender, educational level, degree of dependency, whether the caregivers live at the same home or not, size of the municipality where the patient resides, Autonomous Community, formal in-home care received and formal out-of-home care received. Source: EDAD-08. Analysis using individual terms.

## Discussion

In this study, we estimated that 694.44 million caregiving hours were provided in 2008 in Spain to disabled people that suffer COPD. The translation into monetary figures of this immense amount of care hours ranges from 4,981 to 8,254 million EUR (depending on the shadow price applied). This means that among disabled people with COPD who received informal care, the informal care cost per person ranges from 24,549 to 40,681 EUR per year. This value varies significantly depending on the degree of dependency, ranging from 17,089 EUR per person annually for non-dependents to 33,033 EUR for greatly dependent patients (under the most conservative scenario) and from 28,318 EUR for non-dependents to 54,740 EUR for greatly dependent patients (under the second scenario).

In 2009 total current expenditure derived from dependency care (not just for people with COPD but for all dependency cases) totaled 4,848 million EUR, and in 2010 the figure was 6,767 million EUR [30]. These figures further highlight the great importance of informal care in Spain and the high social cost of COPD.

Generally, the economic impact of COPD is quite high, estimating a total cost that can get up to 1,000 million of euros annually, which of a high percentage is due to direct costs. In fact, hospitalization costs represent between 16% and 43% of the total cost. Indirect costs represent around 60% of the total cost of the illness as the majority of people who suffer from COPD are on working age (around 41%). Furthermore, almost 7% of patient with COPD need care. No study has estimated the economic value of informal care in COPD population as valuating caregiving is quite complicated. However, in this paper we have estimated that a large number of people with COPD need informal care with a very high number of caregiving hours. Because of the prevalence of this disease in the elderly, the total numbers of hours of informal care are quite high. When the entire population is taken into account, and the figures are compared with other chronic diseases such as cancer, stroke, acute myocardial infarction or mental illness, the cost of informal caregiving for people with COPD is significantly higher than the cost of informal caregiving for patients with cancer (1,841.06 million compared with 4,891.18 million annually). Likewise, the cost of informal care given to patients who have suffered from stroke and acute myocardial infarction is slightly lower (5,613.24 and 5,081.62 million EUR, respectively) than in COPD. In contrast, the cost in patients with mental illness is markedly higher (6,600.41 million) compared with those who suffer from COPD [31].

Obviously, the cost of the informal care depends directly on the number of caregiving hours provided. Therefore, identifying which factors explain the variability of these hours is a relevant point that should be taken into account. According to our results, the main factor which explains the variability of caregiving hours is the number of people suffering severe and heavy dependence. Additionally, there are abundant studies on whether formal care provided at home and formal care provided outside the home compensate, replace, reinforce or complement informal caregiving [32-34]. There is widespread agreement that it is convenient to differentiate the types of care because of the varied nature of care involved [35-38]. However, the results and conclusions vary depending on the disease studied, the types of disabilities identified, the formal care services included, and the available data and differences between countries due to cultural and organizational elements. Our results show that formal in-home care complements informal caregiving.

Several limitations of the study should be noted. First of all, the analysis was performed on a sample of disabled people with COPD suffering from disabilities. This means that the results shown cannot be extrapolated to the entire population with COPD. They are only representative of the Spanish population with COPD and related dependencies. Likewise, the existence of a selection bias in the data should be taken into account, because the dependent people analyzed in this study were only those living at home, while all others were excluded. Second, even though the EDAD-2008 provides a wealth of information related to disabled people and their caregivers, it provides cross-sectional and not longitudinal data. This limitation precludes studying the impact of a health shock on the lives of a person and their family. Similarly, because the causal relationships cannot be established between the illnesses suffered and the number of resulting care hours, the figures for the number of care hours cannot be interpreted as being caused exclusively by COPD because other illnesses could impact the number of care hours received. Another final issue that could be considered is the fact that multimorbility could have an important impact on the number of caregiving hours received as it could explain the need for care. Therefore, we checked thought another multivariate regression that once every person who received caregiving with COPD was selected, except for stroke, the rest of comorbidities seemed not to affect significantly on the caregiving hours received. Although the characteristics of the survey does not allow us to determine the clinical stage of people with COPD, this result confirms that people with COPD that need for personal care, require a high amount of caregiving time. The added presence of mental illness, cardiovascular or cancer does not significantly increase the time of care, although it could change the type of care received. The importance of comorbidities in the type of care and its intensity is a field of research that can provide interesting results on the needs of people with COPD and their caregivers.

## Conclusions

The results shown in this paper suggest that, in Spain, any program, strategy or policy of health promotion and care for people with disability cannot overlook the importance of family support. Otherwise, it might constantly face inefficiencies and inequities that would damage the social well-being of both patients and caregivers. Therefore, these results suggest that an integrated approach to the care of dependents needs to take into consideration the role of the primary caregivers. Developing strategies that mitigate the burden undertaken by caregivers would protect informal support, and ensure gains in the welfare of both caregivers and patients.

## Consent

Written informed consent was obtained from the patient for the publication of this report and any accompanying images.
